# Noma: Experiences of Survivors, Opinion Leaders and Healthcare Professionals in Burkina Faso

**DOI:** 10.3390/tropicalmed7070142

**Published:** 2022-07-20

**Authors:** Moubassira Kagoné, Emmanuel Kabengele Mpinga, Marc Dupuis, Marie-Solène Adamou Moussa-Pham, Margaret Leila Srour, Maïna Sani Malam Grema, Ngoyi-Bukonda Zacharie, Denise Baratti-Mayer

**Affiliations:** 1 Nouna Health Research Centre, National Institute of Public Health, Ouagadougou, Burkina Faso; kmoubache@yahoo.fr; 2 Institute of Global Health, Faculty of Medicine, University of Geneva, 1202 Geneva, Switzerland; marie-solene.adamoumoussa@unige.ch (M.-S.A.M.-P.); denise.baratti@unige.ch (D.B.-M.); 3 Institute of Higher Education and Research in Healthcare, Lausanne University Hospital and University of Lausanne, 1010 Lausanne, Switzerland; marc.dupuis@chuv.ch; 4 Health Frontiers, Vientiane, Laos; leila@butterflychildren.org; 5 Faculté des Lettres et Sciences Humaines, University Abdou Moumouni of Niamey, Niamey 10896, Niger; sanigrema@gmail.com; 6 Department of Public Health Sciences, Wichita State University, Wichita, KS 67260, USA; ngoyi.bukonda@shockers.wichita.edu; 7 Faculty of Health Sciences, National Pedagogical University, Kinshasa, Democratic Republic of the Congo; 8 Department of Plastic, Reconstructive and Aesthetic Surgery, Geneva University Hospitals, 1202 Geneva, Switzerland

**Keywords:** noma, experiences, survivors, healthcare professionals, opinion leaders, community

## Abstract

The scientific literature on noma (Cancrum Oris) has clearly increased in recent decades, but there seems to have been limited analysis of issues around the psycho-social impacts of this disease. Even when these issues have been addressed, the focus has tended to be on patient experiences, whereas the community dimension of the disease and the role of healthcare professionals and community leaders in mitigating these impacts remain largely unexplored. A study in the form of semi-directed interviews with 20 noma survivors and 10 healthcare professionals and community leaders was conducted between January and March 2021 in Burkina Faso with the aim of describing the experiences of noma survivors, generating knowledge about living with the burden of the disease and understanding the attitudes of community leaders towards the disease. The results reveal that noma is a disease that affects economically vulnerable populations and leads to extreme household poverty. As far as treatment is concerned, patients tend to turn to practitioners of both traditional and modern medicine. Within communities, noma survivors face discrimination and stigma. The study highlighted a lack of information and knowledge about noma. However, surgical operations lead to patient satisfaction and these remain one of the coping strategies used to tackle the stigma and discrimination. The recommendations set out in this article are aimed firstly at stepping up research into the psycho-social impacts of noma, and secondly at considering these impacts in regional programmes and national plans to combat the disease.

## 1. Introduction

Noma (Cancrum Oris) is a orofacial gangrene known since antiquity [1]. Noma is broadly defined as a pathology that begins with a gingival ulcer which quickly destroys part of the face within a matter of days [2]. From its presence in Europe in the Middle Ages, noma is now found almost exclusively in “developing” countries, especially in sub-Saharan Africa, Asia and Latin America. It primarily affects children aged 2 to 6 who are already suffering from severe chronic malnutrition and other concomitant diseases. It is known in particular for its high mortality rate, which the WHO estimates at being 80–90% in the acute phase [3]. Noma survivors keep with aesthetic and functional sequelae which are a source of suffering, disability and stigma. Other social consequences include fear of contagion and popular beliefs that attribute noma to a curse or spell cast on the child or their family [4,5]. Of note, because noma is present in high-poverty areas, the economic cost of its treatment (i.e., surgery, accommodation) largely overpasses household financial resources [6]. Calls for the recognition of noma as a neglected tropical disease by WHO have emerged in the recent literature [7,8,9].

Noma is wrongly regarded as being a disease exclusive to sub-Saharan Africa. The earliest descriptions of the disease show that noma was present in Europe and in North America [10,11], whereas the recent literature shows that noma is still present in Asia [12].

Predisposing factors for noma include socio-economic factors such as low standard of living, extreme poverty, poor sanitation and proximity to livestock; oral conditions such as poor oral hygiene and the presence of certain forms of gingivitis; systemic conditions such as severe malnutrition, debilitating diseases, malaria, tuberculosis, HIV infection or other immunosuppressive conditions (leukaemia and other blood dyscrasias); and miscellaneous factors such as low birth weight, inadequate weaning, the child’s position within the family at birth and the absence of the mother as the primary caregiver [10,13,14,15]. Current epidemiological data suggests that the global incidence of noma is around 30,000 to 40,000 cases annually with 75% of these occurring in sub-Saharan Africa (the noma belt) [15].

Even though the first descriptions of noma were reported by Hippocrates, the first thesis on the disease dates back to 1848 and the first Medline article was published in 1852 [16], little is known about the disease and research into the condition is only relatively recent. From a timeline perspective, research on noma was relatively modest, with one article published in 1901 on Medline and only one other article in 1960 on the same database. The turning point seems to have been around the turn of the century. In 2003, 14 articles were published, but by 2020, this figure had more than doubled to 34 [7].

Regarding the subjects tackled in these publications, it is evident from the literature that these have evolved over time. The earliest articles focus on the main clinical characteristics and treatment of the disease and are generally case studies [17,18]. Later publications describe surgical techniques [19,20,21].

Much rarer and far more recent are research articles aimed at shedding light on the epidemiology or aetiology of this disease. On this latter point, various theories have been expounded, such as vitamin and micronutrient deficiencies or virological and/or bacterial theories. Only two case-control studies have been published that aim to identify risk factors associated with noma [15,22].

Over the years, various literature reviews have been published: firstly, the freely available review by the Ashok group, which covers the period from 2003–2014. The review found an increased incidence of noma among HIV-positive patients [13].

Three systematic reviews were later published [7,8,23]. In brief, most of articles were published in the 2000s and focused on medical aspects of the diseases (aetiology, treatment) [7,8]. Resulting from a systematic review, at least 23 countries with noma cases were identified with most of cases in the noma belt counties in West Africa [23]. Overall, the literature clearly shows that progress has been made in terms of knowledge and prevention of the disease but also highlights major gaps, some of which have been identified in the research project of which this study is a part (i.e., the noma project). Two of the gaps identified are a lack of accurate and up-to-date epidemiological data on noma, and a lack of understanding of the experiences of persons at risk and survivors of noma.

Regarding this latter point, although the stigma and discrimination experienced by direct and indirect survivors of noma is recognised as having an enormous burden on the survivors themselves and on communities, there is little or no knowledge of the experiences of survivors, opinion leaders and healthcare professionals.

Based on 15 relevant articles published between 2006 and 2015, Wali and Regmi’s review of the literature to establish existing knowledge about social stigma associated with noma-related facial disfigurements identified four categories of studies based on their themes. These are (i) manifestations of social stigma, (ii) consequences of stigma within the individual, (iii) coping with the stigma and (iv) positive social influences on coping [24]. These results indicate that the experiences reported are those of noma survivors alone and do not consider the opinions and attitudes of other stakeholders involved in noma prevention and control, such as healthcare professionals and community leaders.

To fill this gap, the present study aimed to interview noma survivors, healthcare professionals and opinion leaders to compare their experience and knowledge about the burden of noma and have a deep understanding of the community attitudes towards both the diseases and its survivors.

This knowledge is both useful and necessary for public health policy makers, planners, financial backers and various stakeholders interested in improving the living conditions of patients with noma and noma survivors as well as for the households and communities in which these patients and survivors live.

## 2. Context of the Study

The study was conducted in Burkina Faso. Located in the heart of West Africa, Burkina Faso covers an area of 274,200 km^2^. It is bordered to the North and West by Mali, to the East by Niger and to the South by Benin, Togo, Ghana and the Ivory Coast.

The country is divided into 13 regions, 351 communes (49 urban and 302 rural) and 8228 villages. The regions and the communes are local authorities with legal personality and financial autonomy.

In 2010, the country’s population was estimated at 15,730,977 and women represented 51.7% of the overall population [25]. Most of the population (77.3%) lives in rural areas living from agriculture and stock farming. The crude birth rate was 46 per 1000. The population is predominantly young. The 0–15 age group represents 46.4% of the population. The total fertility rate is 6.2 for the country as a whole [26].

With a gross domestic product per capita of USD 790 in 2019, Burkina Faso is among the countries that produce the least wealth per capita. According to World Bank figures, in 2014, 43.8% of the local population lived below the national poverty line. According to a United Nations Development Programme report, the country ranks 182 out of 189 countries with a Human Development Index of 0.45 in 2020 [27,28].

The health system in Burkina Faso largely relies on many private healthcare facilities [27] that are mostly located in the cities of Ouagadougou and Bobo-Dioulasso, leaving rural populations potentially far from adequate healthcare structures, especially to treat noma. In 2019, there were 593 private healthcare facilities [29].

## 3. Methods

### 3.1. Study Proposal

This was a qualitative study in the form of semi-structured interviews. The interview guide was developed in conjunction with team members in Burkina Faso and Niger. It was then tested and adjusted through pilot interviews in Burkina Faso and Niger before the start of data collection. Substantive and editorial corrections were made to the original version.

### 3.2. Study Design and Sample

The study sample consists primarily of patients who have suffered from noma. The patients could be adult, but adolescents and children were also eligible. Of note, they could either have undergone or be awaiting surgery and care from the *Sentinelles foundation*. Being in the acute stage of noma (or of other diseases) represented the only exclusion criterion (i.e., such a state requiring a quick transfer to a hospital).

Secondly, the study includes opinion leaders who have an idea about the disease and its consequences. This second group consisted of representatives of public authorities (i.e., religious, political) at a local level, and of teachers and healthcare professionals playing a first-line role.

The actual number of respondents depended on the saturation of information obtained during the data collection stage. Opinion leaders were selected on rational grounds rather than on a random basis (i.e., due to their role, they had much influence on the community).

### 3.3. Data Collection and Analysis

Data collection and analysis took place in the period from January to March 2021. This stage was managed by an anthropologist with a background in both social sciences and public health. The anthropologist is from the country, has years of professional experience in the field and speaks several local languages. A semi-structured interview guide was used for each type of participant. The interviews were conducted in Dyula, Mooré (local languages spoken in the study area) and French. The length of the interviews was between 30 and 45 min. The interviews were recorded after each participant (or their representatives) had given their informed consent. The recordings were then transcribed. The resulting transcripts were managed using the qualitative analysis software NVivo PRO 12 (QSR International Pty Ltd., Victoria, Australia, https://www.qsrinternational.com/nvivo-qualitative-data-analysis-software/home, accessed on 22 August 2021).

Thematic and content analysis approaches were used to process the data based on Braun and Clarke’s approach [30]. Thematic coding procedures were established, and a list of codes generated to guide the analysis. This list was updated as the analysis progressed.

### 3.4. Ethical Considerations

Informed consent was sought from all participants prior to their participation in the study. Each potential participant was given appropriate information about the objectives, methods, expected benefits and risks of the study, as well as about any embarrassment it might cause. Participants were also informed of their right not to participate in the study or to withdraw their consent to participate at any time without repercussions. Patients aged below 18 years of age could participate subject to the written consent of their parent or legal representative. Additionally, child participants could be accompanied by one parent. Consents were conserved in a safe place in Burkina Faso, separate from the collected data that were coded and transferred to the project headquarters in Geneva.

Confidentiality was strictly observed. The interviews took place in convenient locations where participants could be reassured that their privacy and confidentiality could be maintained. All raw data remains confidential and is accessible only to the research team. No individuals can be identified in the results distributed or in the reports related to this study. Ethical approval was obtained from Burkina Faso’s National Health Research Ethics Committee (no. 2020.6.106).

## 4. Results

A total of 30 persons were interviewed until reaching saturation. The sample included 20 noma survivors and 10 opinion leaders. Half of the survivors were still aged below 15 years of age. One of them was only 5 years old, which was much younger than the other participants and required the presence of the mother. The sample was gender-balanced, with 11 (55%) participants being male. Most of them (17, 85%) were living in rural places.

Regarding opinion leaders, three were local political authorities, three were religious authorities (one imam, one priest and one reverend, so that each main religion was represented), two teachers and one local health agent were enrolled.

### 4.1. Knowledge of the Disease in the Community

In Dyula, noma is called “*Bamba-dimi*”, where *bamba* means “crocodile” and *dimi* means “*pain*”. This terminology suggests that the pain of noma is like that of a crocodile bite.

Most of the resource persons we interviewed knew about the disease because they had heard about it or seen images of it on TV or knew close relations who had been affected by the disease.

“*Well, I don’t actually know much about Noma, but I have heard about it at least. In Dyula, noma is called Bamba-dimi. I’ve heard of it before and I’ve even come across a number of Noma patients*.” (Leader 1: Municipal Councillor)

Confusion and misconceptions exist about noma:

“*What I know is that its other name is harelip. In the cases I’ve seen, not directly but perhaps on TV, it usually affects children. That’s about it. I also know that there is a team, based in Ouahigouya I think, which cares for those who have been affected by the disease. That’s it. That’s all I know*.” (Leader 2: Teacher)

Although harelip is not the same issue as noma, the last is clearly identified as an orofacial problem.

The known signs or symptoms of the disease are sores in the mouth and cheek. According to resource persons, noma survivors in the community generally report pity, suffering or disgust. Patients regularly hide and protect their face. The difficulties of providing care in certain locations have fatal consequences.

“*Well, I once knew a Noma patient. He was a school teacher, his disease started with swelling of his cheeks and his eyes were affected. He was often forced to protect his mouth. At one point his lips were shredded, eaten away. He turned to modern medicine and traditional care but it was all in vain. The disease lasted for about four years. Afterwards, the man in question died. He lost a lot of things, particularly as a result of this disease*.” (Leader 3: Municipal Councillor).

### 4.2. Onset of the Disease

Most patients report that the initial symptoms of the disease are dental pain, spots on the nose and sores in the mouth and nose. In the majority of cases, the disease affects children under the age of two. The following regularly repeated comments support these ideas.

“*Yes, it started with a sore on his mouth; over time, the sore spread to his nostrils and continued just like that*.” (Patient 10).

“*According to her [i.e., the patient’s mother], it has now been seven years since it started. They themselves thought it was perhaps toothache*.” (Patient 1).

“*The truth is that the disease started when I was very young. So, I was too young to know anything about it*.“ (Patient 16).

### 4.3. Course of the Disease

The course of the disease is almost identical in the majority of patients. It is a disease that is unknown to patients before it occurs and even unknown to the primary local healthcare facilities. It starts with facial sores. Patients visit the nearest health centres for care. These community clinics or other health centres have limited knowledge and resources and the disease typically continues to worsen. Patients are referred to the next level of care. It is often at the next level that a proper diagnosis is made. By that time the patient has already suffered a lot and lost time. The story of a 43-year-old patient is a perfect illustration of this:

“*The start of my illness was Noma. I don’t know the name in Mooré. As it progressed, it consumed my lips until it reached my nostrils, to the point where I could no longer breathe. It was at that moment that I was transferred from Kongoussi hospital to Yalgado (Ouagadougou University Hospital). […] So, it was at Yalgado that I received treatment. The wound healed but the cleft is still there. I’ve been like this for a whole year. […] One day we met the people from Sentinelles in Kongoussi and a nurse explained my case to them. They came to see me and asked me to come here (to Sentinelles in Ouagadougou) so they could help me repair my nose. That’s how they helped me*.” (Patient 11).

### 4.4. Treatment Options for Patients with Noma

Persons suffering from noma have two options: modern and traditional care. It should be said that at the onset of the disease, most patients firstly turn to traditional care but this does not result in any improvement. Then they turn to modern treatments and stop bothering with traditional products.

“*Yes, at the time of the disease we used traditional treatments. It’s true! We first turned to traditional care. We would apply these traditional products onto the wound. The next morning, when I woke up, I could see that the product had dried up the wound and torn off the flesh, which just fell off like that. […] It was only when I arrived at Yalgado and received treatment there that we saw an improvement*.“ (Patient 11).

For noma sufferers, traditional care does not lead to any improvement in the disease. The reason why households affected by this disease resort to traditional care or to prayer is simply due to poverty.

“*At this time, my parents did not have the financial resources to heal me. There were products against the illness, but they missed the resources to buy them. Eventually, my sake was left in God’s hands. They did not even think that I would survive. Before the illness was gone, it had taken one side of my lips*.” (Patient 1).

Accordingly, patients have access to modern treatments only after the failure of the traditional healers. One opinion leader commented:

“*As I’ve said, for instance, if parents don’t even have resources to pay for healthcare or for surgery… well, it’ll stay like that*.” (Leader 5: Reverand)

Finally, comorbidities can lead to situations where surgical treatment must be postponed, even when the patient arrives quickly to receive medical treatment:

“*When we arrived where they treat people, it was found that there was also another illness than this one. We spent 21 days there before benefitting from this treatment. The cured the other illness first before surgery*.” (Patient 6, benefitted from surgery during the acute stage of noma).

### 4.5. Information about the Operation

The patients interviewed were very happy with the operation carried out to rectify the consequences of the disease. Surgery improves the aesthetic appearance of noma survivors, solving the problem of shame when appearing in public. In addition, patients acknowledge that the operations are free of charge and carried out by the Sentinelles foundation. The success of the operations carried out by Sentinelles encourages patients to undergo surgery.

“*No, in my opinion there are no concerns. They are the doctors and they know what to do to make things better. And what’s more, it’s for my own well-being! So, I don’t see why I wouldn’t welcome this information. If someone helps you the first time and you have seen the results, if that same person comes back to complete what they have started, I don’t see any problem with that. In fact, it is a blessing for you, a gift from God. If it’s not a burden for the surgeon, why would I refuse to accept his help! So, no problem there*.“ (Patient 17)

“So, when I heard that they can fix it, I came. And if it works, I’d be very happy. “ (Patient 3, awaiting treatment).

### 4.6. Reactions after Surgery

After surgery, patients and their escorts are happy with the results and most of them express their delight. For some patients, surgery restores their pride and joy of living.

“*I was really happy. After the operation, the very people who had been nasty to me were the first to come and wish me well. […] From that moment on, I started to regain my pride, my joy of living, and this is increasing day by day*.” (Patient 11)

“*Reconstructive surgery is not easy, it must be very expensive. But it’s a very, very good thing to give these people much needed relief. It brings a great ray of hope. […]. That’s it! It really makes patients feel better about themselves. So, I think it makes good sense to be able to repair these disfigured faces*.” (Leader 8: Community priest).

### 4.7. Lack of Information about the Disease

Patients are not sufficiently informed about the disease. Most of them do not know much about the disease. Knowledge of noma among health workers outside of the major towns and cities is insufficient. As the consequence, they cannot properly diagnose the disease. One patient explains how he was treated at a local hospital:

“*So, they went to show that there is…, a thing…, spot on the nose but it swells, if you touch it, it hurts too. We were given…, a…, a thing to go and buy medicines. So, we went to buy, they put injections, linctuses. And after…, well! And two (2) days later, the thing, the wound started to rot*.” (Patient 8).

Even when health workers are able to identify noma, they lack knowledge of its aetiology. For instance, though noma is not hereditary, one health professional states:

“*Noma is a congenital malformation. It occurs in infants from the age of two onwards after a sudden weaning, malnutrition, because the immune system is deficient. […] There are factors that can bring about…, which can make cases of noma occur*.” (Leader 4: Health professional).

The inadequacy of care in health centres outside of the major towns and cities was underlined by the respondents:

“*We felt helpless, we didn’t understand. We don’t know anything about this disease, we didn’t receive any information about it. We felt that it was simply down to God*.” (Patient 1).

### 4.8. Experiences of the Disease in the Community: Discrimination and Stigma

Most patients experience discrimination and stigma. In the community, noma survivors are pointed at, mocked and insulted by friends and even by the family members who are supposed to protect them. For these patients, this situation leads to psychological suffering in addition to the physical suffering caused by the after-effects of the disease.

One respondent said:

“*It’s all because of the disease. It was the disease that caused my paralysis. Whenever I go into a place where there are a lot of people, they make fun of me. They often gesture and point at me, and that also causes me pain*.” (Patient 11).

Feelings of disgust and mistrust of patients are also reported. Some community members feel that people living with noma should not mix in crowds. They should stay away.

“*There are some, if they are heads of households, they have to say that in their village, they see people who don’t have a healthy body and then they go among the people. So, if you hear that, you won’t be happy*.“ (Patient 14).

“*There’s no doubt that if someone is in this situation, besides your loved ones, many people hate you and find you disgusting. You even find yourself disgusting. For example, if you are eating in the company of other people, even if we’re not using the same container and each person has their own plate, some don’t like the fact that you’re sitting with them at the same table*. “(Leader 6: Imam, community leader).

The public’s critical view of noma survivors makes life unpleasant and an unbearable ordeal for them.

“*When you are the only one in the village with this disease, you become the village pariah. The stigma drives you crazy and you suffer an unbearable ordeal*.” (Patient 15).

Married patients experience negative criticism and rejection often from the spouse’s family, especially women. This comes in the form of insults about the disability caused by the disease. For example:

“*They were saying that instead of him [the husband] going to look for a perfect wife, he should go to find a woman who has a split nose*.” (Patient 11).

It is not only married survivors who are insulted. Most unmarried survivors acknowledge that it is hard to find a spouse because of the consequences of the disease. Noma survivors experience difficulties in finding a partner. One respondent confirmed this:

“*All the women I have courted have rejected my advances. They even say that although it’s not me, they have never seen anyone else with this kind of disease. Apart from my parents, many people wouldn’t even come near me*.” (Patient 15).

Because of the disease, some patients refuse to be photographed or to look at themselves in the mirror. The fear of looking at a face with disfiguring scars prompts patients to avoid anything that may reflect their image: “Photo booths were a no-go area for me” (Patient 11).

The stigma attached to criticism and the way members of the community look at noma survivors leads to withdrawal and self-isolation. Patients no longer participate in community events such as family gatherings, baptisms or any gathering of people.

“*I have found it tough because since my nose split, even being allowed to be among other people has been impossible. […] Even within my own family, if there is a gathering of family members, I wouldn’t take part*.” (Patient 11)

Some patients report that they want to commit suicide in the face of the rejection, suffering and humiliation felt when coming into contact with lots of people:

“*I was even contemplating suicide. Because I was treated as if I were not a human being. I was different from others. When I looked at myself in the mirror, I lost faith in God. I often thought of killing myself*.” (Patient 15).

“*The victims themselves find that life is no longer important to them, not to mention others. When you can’t be around people, you feel disgusting and hated. You can’t eat or drink with others from the same container. As the person who knows your own situation, you start to hold yourself back and keep out of the way before you are criticized*.” (Leader 6: Imam, community leader).

### 4.9. Coping Strategy/Solution to the Problems of Discrimination, Stigma and Withdrawal

Most patients feel that surgery offers a solution to the discrimination and stigma they experience as it helps to repair the consequences of the disease on their face. Surgery allows patients to feel reborn with a new face. The words of this patient who has a new face after surgery illustrate this point.

“*After treatment I became a new person. I got married and I’ve had two beautiful children. I could never imagine that one day I would have a wife, let alone have children*.” (Patient 15)

As well as surgery, some patients are helping to raise awareness of the disease and, in particular, the fact that noma is not contagious. This work to raise awareness in the community helps to reduce the fear of contagion.

“*They say that your illness will contaminate them […] Now it’s up to you to help them understand that the disease you had is not contagious*.” (Patient 13)

Some patients, in order to avoid stigma and discrimination, use masks or scarves to hide or cover the disability before entering a crowd:

“*When I went to school, there were other friends who would make fun of me. So, I would tie my scarf over my head. And hide my face*.” (Patient 13)

Many patients look for solutions to make their scars disappear. These include ointments or medicines sold on the market.

“*Yes, I’ve spent money, because..., from time to time, some of my friends would tell me that there are products that you can rub on and your scars will disappear instantly. […] So, I tried to buy some of these products. […] My goodness, the prices! I’ve tried several times. For some products, I’ve paid 3500, 7000, even more. For others, I’ve I paid 11,000*.” (Patient 10)

Some patients turn to their faith to help explain and accept their condition as a means of developing resilience in accepting the disease and its consequences. No matter what the reaction of community members is, everything must be left in the hands of the Lord.

“*So, if it’s something that belongs to you, something that’s within you, it doesn’t matter what people say. You leave everything in God’s hands*.” (Patient 15)

## 5. Discussion

The results of our study corroborate those of other studies conducted on stigmatising diseases such as the experiences of survivors of leprosy and HIV. The loss of social worth due to the scarring, exclusion and suffering caused by the effects of treatment or the disease itself impacts the course of the disease and diminishes quality of life for those who are HIV-positive [31].

Similar conclusions were found regarding leprosy patients who were isolated and abstained from various family activities. Although both men and women are affected in terms of their social lives, women suffer more isolation and rejection [32]. Gender differences regarding discrimination among noma survivors were however little discussed in the present study. Another similarity with leprosy concerns the fact that negative community behaviors are motived by the fear of a curse from God [33].

Returning to noma and the complexity of its social consequences, the study by Brattström-Stolt et al. in Burkina Faso emphasises that noma is a disease that particularly impacts economically vulnerable populations. It leads to extreme poverty. Household property is sold off to cope with the costs of the disease, and income also diminishes because carers can no longer go about their business [34].

Persons suffering from noma tend to turn to practitioners of both traditional and modern medicine. Patients living in rural areas are often late in discovering the disease because local health centres are unable to diagnose noma cases in time [2,35]. Ignorance of the early symptoms of noma is mentioned in our study as being a factor limiting swift patient management.

As for delays in seeking treatment, the average delays remain long and undoubtedly explain the high levels of morbidity and mortality linked to noma, as well as the difficulty of knowing the real prevalence of the disease. Some families turn to traditional treatment using, among other things, applications that involve plants or animal dung. According to Tall and colleagues [36], 95% of the children arriving at the hospital of Bobo-Dioulasso with acute noma had received one of these traditional treatments first, which resulted in average consultation time of about 18 days with a range of 5 to 56 days.

Lack of awareness of noma is an important factor highlighted by our study both among patients and healthcare workers outside of the major towns and cities. This lack of information and knowledge is also highlighted by Brattström-Stolt and colleagues [34].

Inadequate noma training for front-line healthcare workers is another factor that accounts for both the lack of awareness of this disease and its prevalence. In its integrated strategy for fighting non-communicable diseases, the Ministry of Health points out that there is a lack of human resources with expertise in managing these conditions in Burkina Faso [37].

The coping strategies used by patients to deal with the stigma and discrimination are facial reconstructive surgery or the use of scarves or masks to hide the disability. These strategies are not dissimilar to those adopted by patients with cleft lip defects in Nigeria [38]. The patient satisfaction reported in our study is not specific to the population studied or to the disease in question. It is characteristic of relationships of help, including relationships of care [39].

Our study provides a better understanding of the suffering of patients with noma and noma survivors by highlighting the difficulties of managing cases. Extreme poverty favours the development of the disease while being exacerbated by the disease itself. It turns into a vicious cycle of “poverty-illness-poverty” tending to make the condition endemic. The expression “*the face of poverty*” coined by the WHO and used by others in relation to noma is therefore fully justified [11,40].

Beyond the external validity of these results, our study presents the following strengths. From a technical point of view, the patients included in this study were interviewed in different environments (living spaces and the Sentinelles foundation’s care centre), and at different stages of the disease (patients awaiting surgery, patients who have already undergone surgery, former patients who have lived with the disease for a long time).

Secondly, their experiences, opinions and perceptions of the problems were taken into account as part of a participatory approach, which demonstrates our concern to reinforce the right to health (patients’ rights) by respecting their freedom of expression and opinion.

Finally, one of the distinctive features of this study is that it combines the perceptions and attitudes of healthcare professionals and community leaders with patient opinions. Although at this stage the results of a comparative analysis of the various opinions of these categories of participants have not been released (they will be analysed subsequently), the perspective used reveals a convergence of views.

Despite these strengths, the results of this study need to be seen within their contextual and cultural limitations. The study was conducted in a sub-Saharan African country with a high level of social stratification, with its own individual organisation of its health system and delivery of healthcare, all of which has a bearing on life, illness and death.

Because some of the patients did not speak the country’s official language (French), we needed to translate their comments from Dyula or Mooré into French. The transcriptions and analyses carried out in French may have altered somewhat the content of the responses given by the participants.

Finally, for patients who suffered from the disease in the relatively distant past, recall bias cannot be ruled out.

Despite these limitations, the results of our study mean that we can make a number of recommendations in terms of research and action to combat noma.

From a research perspective, we think it would be useful to:

- Develop and extend the studies into the psycho-social and economic impacts of noma;
- Incorporate the results of the impact studies into the regional plans and national programmes to prevent and combat noma;
- Extend these impact studies to the communities by including the perspectives of healthcare professionals, educators, policy makers and other opinion leaders;
- Promote teaching and research on noma in training schools for healthcare professionals.
- In terms of possible interventions, these should include:
- Increasing skills among front-line health workers as regards early detection and management of acute noma;
- Incorporating psycho-social elements into the care provided for noma survivors;
- Training healthcare professionals in the surgical management of patients with noma;
- Raising awareness among community leaders about the conditions under which this disease emerges and spreads.

## 6. Conclusions

The objectives of this study were to describe the experiences of noma survivors, to generate knowledge about living with the burden of the disease and to understand the attitudes of community leaders towards the disease.

The results show that noma is a disease that affects economically vulnerable populations and leads to extreme household poverty. Persons suffering from noma tend to seek care from practitioners of both traditional and modern medicine. Within the community, noma survivors face discrimination and stigma. The study also highlighted a lack of information and knowledge about noma. However, patient satisfaction after surgery remains one of the coping strategies used to tackle the stigma and discrimination.

The results of the study will help to raise awareness among policy makers of the need for greater involvement of community members in supporting patients. Similarly, increased awareness of the disease among the population will improve early detection and ensure that appropriate treatment is made available in order to reduce avoidable patient death, facial sequalae and related social discrimination.

## Data Availability

Some of the data mainly used in this article come from the Sentinelles Foundation database sheet. Any request to use these data should be made with the agreement of this Foundation.

## Appendix A

List of Funders and Partners of the Noma Project.

1. Lead Organizations are the University of Geneva, Switzerland; The University of York, UK; The Swiss Tropical and Public Health Institute, Basel, Switzerland.
2. Funding Organisations are the Swiss network for international studies, Geneva, Switzerland; Hilfsaktion Noma e.V. Regensburg, Germany; Service de la Solidarité Internationale Geneva, Switzerland; Noma-Hilfe-Schweiz, Zurich–Switzerland; Winds of Hope, Lausanne, Switzerland.
3. Partners Organisations are Foundation Sentinelles, Lausanne, Switzerland; Health Frontiers Laos, Vientiane, Laos; Médecins Sans Frontières, Geneva, Switzerland; SongES, Niamey, Niger; International No Noma Federation, Lausanne, Switzerland.
4. Academics Partners are the Centre Interfacultaire en Droits de l’Enfant, University of Geneva, Switzerland; Centre de recherche en santé, Burkina Faso; Geneva Health Forum, Geneva, Switzerland.
5. Governmental Bodies are the Ministry of Health, Ouagadougou, Burkina Faso.
6. Programme National de lutte contre les maladies bucco-dentaires et le noma, Nouakchott, Niger.
7. Intergovernmental Partners are the United Nations Human Rights Council Advisory Committee; the United Nations Children’s Fund (UNICEF), Niger; the World Health Organisation, Geneva.

**See the Noma Project Web Site:**https://thenomaproject.org/partners (accessed on 18 February 2022).

## References

[1] Marck K.W. (2003). Cancrum oris and noma: Some etymological and historical remarks. Br. J. Plast. Surg..

[2] Baratti-Mayer D., Pittet B., Montandon D., Bolivar I., Bornand J.-E., Hugonnet S., Jaquinet A., Schrenzel J., Pittet D. (2003). Noma: An “infectious” disease of unknown aetiology. Lancet Infect. Dis..

[3] WHO (2013). Atelier Inter-Pays sur le Programme Régional de Lutte Contre le Noma.

[4] Srour M.L., Marck K.W., Baratti-Mayer D. (2015). Noma: Neglected, forgotten and a human rights issue. Int. Health.

[5] Srour M.L., Marck K., Baratti-Mayer D. (2017). Noma: Overview of a Neglected Disease and Human Rights Violation. Am. J. Trop. Med. Hyg..

[6] Mpinga E.K., Srour M.L., Moussa M.-S.A., Dupuis M., Kagoné M., Grema M.S.M., Zacharie N.-B., Baratti-Mayer D. (2022). Economic and Social Costs of Noma: Design and Application of an Estimation Model to Niger and Burkina Faso. Trop. Med. Infect. Dis..

[7] Dominic C., Farley E., Elkheir N. (2022). More than 100 years of neglect: A bibliometric analysis of global research on noma (cancrum oris). Trans. R. Soc. Trop. Med. Hyg..

[8] Farley E., Mehta U., Srour M.L., Lenglet A. (2021). Noma (cancrum oris): A scoping literature review of a neglected disease (1843 to 2021). PLoS Negl. Trop. Dis..

[9] Srour M.L., Baratti-Mayer D. (2020). Why is noma a neglected-neglected tropical disease?. PLoS Negl. Trop. Dis..

[10] Ogbureke K.U., Ogbureke E.I. (2010). Noma: A Preventable “Scourge” of African Children. Open Dent. J..

[11] Marck K.W. (2003). A history of noma, the “Face of Poverty”. Plast. Reconstr. Surg..

[12] Srour M.L., Farley E., Mpinga E.K. (2022). Lao Noma Survivors: A Case Series, 2002–2020. Am. J. Trop. Med. Hyg..

[13] Ashok N., Tarakji B., Darwish S., Rodrigues J.C., Altamimi M.A. (2015). A Review on Noma: A Recent Update. Glob. J. Health Sci..

[14] Pedro K., Smit D.A., Morkel J.A. (2016). Cancrum Oris (noma) in an HIV-positive adult: A case report and literature review. South Afr. Dent. J..

[15] Farley E., Lenglet A., Ariti C., Jiya N.M., Adetunji A.S., Van Der Kam S., Bil K. (2018). Risk factors for diagnosed noma in northwest Nigeria: A case-control study, 2017. PLoS Negl. Trop. Dis..

[16] Tonna J., Lewin M.R., Mensh B. (2010). A case and review of noma. PLoS Negl. Trop. Dis..

[17] Mackay D. (1949). Cancrum oris among African natives. Br. Med. J..

[18] Tempest M.N. (1971). Cancrum oris. Trop. Dr..

[19] Pittet B., Jaquinet A., Montandon D. (2001). Clinical experience in the treatment of noma sequelae. J. Craniofacial Surg..

[20] Marck K.W., De Bruijn H.P. (1999). Surgical treatment of noma. Oral Dis..

[21] Montandon D., Lehmann C., Chami N. (1991). The surgical treatment of noma. Plast. Reconstr. Surg..

[22] Baratti-Mayer D., Gayet-Ageron A., Hugonnet S., François P., Pittet-Cuenod B., Huyghe A., Bornand J.-E., Gervaix A., Montandon D., Schrenzel J. (2013). Risk factors for noma disease: A 6-year, prospective, matched case-control study in Niger. Lancet Glob. Health.

[23] Galli A., Brugger C., Fürst T., Monnier N., Winkler M.S., Steinmann P. (2022). Prevalence, incidence, and reported global distribution of noma: A systematic literature review. Lancet Infect. Dis..

[24] Wali I.M., Regmi K. (2017). People living with facial disfigurement after having had noma disease: A systematic review of the literature. J. Health Psychol..

[25] Institut National de la Statistique et de la Démographie (2009). Annuaire Statistique.

[26] Population Data (2021). Burkina Faso. https://en.populationdata.net/countries/burkina-faso/.

[27] Ministère de la Santé (2011). Plan National de Développement Sanitaire 2011–2020.

[28] UNDP (2020). The Next Frontier. Human Development and the Anthropocene.

[29] Ministère de la Santé (2021). Annuaire Statistique 2020.

[30] Braun V., Clarke V. (2006). Using thematic analysis in psychology. Qual. Res. Psychol..

[31] Van Rie A., Sengupta S., Pungrassami P., Balthip Q., Choonuan S., Kasetjaroen Y., Strauss R.P., Chongsuvivatwong V. (2008). Measuring stigma associated with tuberculosis and HIV/AIDS in southern Thailand: Exploratory and confirmatory factor analyses of two new scales. Trop. Med. Int. Health TM IH.

[32] Zodpey S.P., Tiwari R.R., Salodkar A.D. (2000). Gender differentials in the social and family life of leprosy patients. Lepr. Rev..

[33] de Stigter D.H., de Geus L., Heynders M.L. (2000). Leprosy: Between acceptance and segregation. Community behaviour towards persons affected by leprosy in eastern Nepal. Lepr. Rev..

[34] Brattström-Stolt L., Funk T., Sié A., Ndiaye C., Alfvén T. (2019). Noma-knowledge and practice competence among primary healthcare workers: A cross-sectional study in Burkina Faso. Int. Health.

[35] Baratti-Mayer D., Baba Daou M., Gayet-Ageron A., Jeannot E., Pittet-Cuenod B. (2019). Sociodemographic Characteristics of Traditional Healers and Their Knowledge of Noma: A Descriptive Survey in Three Regions of Mali. Int. J. Environ. Res. Public Health.

[36] Tall F., Ki-Zerbo G., Ouedraogo I., Guigma Y. (2001). Le noma de l’enfant en milieu hospitalier de Bobo-Dioulasso: Aspects épidémiologiques, cliniques et prise en charge. Odonto-Stomatol. Trop..

[37] Ministère de la Santé (2013). Plan Stratégique Intégré de Lutte Contre les Maladies non Transmissibles 2014–2018.

[38] Umweni A., Agbontaen-Eghafona K., Sauter H. (2009). Social analysis of cleft lip and palate abnormality in Nigeria. J. Soc. Behav. Health Sci..

[39] Mpinga E.K., Chastonay P. (2011). Patient Satisfaction Studies and the Monitoring of the Right to Health: Some Thoughts Based on a Review of the Literature. Glob. J. Health Sci..

[40] Leclercq M.-H., World Health Organisation (1999). The Face of Poverty. Noma Contact: Cancrum Oris Network Action.

